# Correlation between Pre- and Post-Surgical Findings for Long-Term Neurocognitive and Behaviour Development Due to Posterior Fossa Pilocytic Astrocytomas: The Trend after 10 Years

**DOI:** 10.3390/diagnostics11081489

**Published:** 2021-08-17

**Authors:** Daniela Pia Rosaria Chieffo, Valentina Arcangeli, Federica Moriconi, Camilla Zanetti, Paolo Frassanito, Federico Bianchi, Luca Massimi, Gianpiero Tamburrini

**Affiliations:** 1 Clinical Psychology Unit, Fondazione Policlinico Universitario Agostino Gemelli IRCCS, 00168 Rome, Italy; danielapiarosaria.chieffo@policlinicogemelli.it (D.P.R.C.); valentina.arca@gmail.com (V.A.); fefes.moriconi@gmail.com (F.M.); cami.znt@gmail.com (C.Z.); 2 UOC Neurochirurgia Infantile, Dipartimento di Scienze Dell’Invecchiamento, Neurologiche, Ortopediche e della Testa-Collo, Fondazione Policlinico Universitario A. Gemelli—IRCCS, Università Cattolica del Sacro Cuore, 00168 Roma, Italy; luca.massimi@policlinicogemelli.it (L.M.); gianpiero.tamburrini@unicatt.it (G.T.); 3 Pediatric Neurosurgery Unit, Fondazione Policlinico Universitario Agostino Gemelli IRCCS, 00168 Rome, Italy; paolo.frassanito@policlinicogemelli.it

**Keywords:** brain tumour, neurocognitive, surgical treatment, pilocytic astrocytoma

## Abstract

Objective: The objective of the present study was to selectively evaluate the long-term impact of posterior fossa pilocytic astrocytomas, which are known to be among the most benign forms of paediatric brain tumours on neurocognitive and behavioural functions. Methods: Children that were operated on for a posterior fossa pilocytic astrocytoma in the Pediatric Neurosurgery Department of the Catholic University Medical School were selected according to the following criteria: (a) age > 5 years (in order to have a complete set of neurocognitive evaluations data), (b) ability to perform a complete set of tests before and after surgery, and (c) children that had a regular follow-up up to 10 years from the surgical treatment. Results: Forty-three percent of the children selected for the present study showed a borderline IQ before surgery, which is a result corresponding to those previously reported in the literature for children affected by posterior fossa pilocytic astrocytomas; praxis and visual perception were the selective functions that were more frequently affected. Language performance tests scores were below average in 40% of the cases but tended to improve in terms of expressive and receptive skills even at the 1-year follow-up; the improvements became significant at the 5-year and 10-year follow-ups. Conclusions: Recognising and measuring the short- and long-term effects of cerebellar tumours in children and their treatment are the first step towards improving their clinical course and quality of life. Early interventions should be offered to all of them, with specific attention bestowed on visual-spatial stimulation, speech and occupational therapies in order to act on praxic and visuo-perceptive skills, as well as on emotion and behaviour tracts of the neurocognitive profile, which more commonly tend to persist in the long term.

## 1. Introduction

Factors that have been recognised as possibly influencing neurocognitive development in children with brain tumours before surgery are: tumour localisation, the presence of altered neurocognitive functions at diagnosis and associated hydrocephalus. Postoperatively, the tumour grade and adjuvant treatments (namely, chemotherapy and/or radiotherapy) are the most common factors that are related to the persistence and/or worsening of patients’ neurocognitive status [1].

Even though a progressive recovery of pre- and post-operative deficits can usually be expected, there are no clear data on how long such a recovery can continue or the factors contributing to a lack of improvement [2]. Such a rule is also true for pilocytic astrocytomas, which, although they are considered cured after the tumour removal, might exhibit persistence of defined selective neurocognitive function impairment. When posterior fossa pilocytic astrocytomas are specifically considered, deficits in executive functions, mood instability and language difficulties, as well as compromised spatial cognition and mood regulation.were documented. Further, deficits in attention [3,4,5,6] processing speed [4] and memory were also observed [4]. Advising early rehabilitation to consolidate and improve patients’ recovery is of utmost importance, as is universally agreed in the existing literature. Nonetheless, there are no studies with a long follow-up to support the aforementioned statement.

The present study aimed to evaluate how long the improvement of the neurocognitive functions of children with posterior fossa astrocytomas may continue in a population that underwent a comprehensive neurocognitive and behavioural assessment before surgery and after tumour removal in a staged follow-up lasting up to 10 years from surgical treatment.

## 2. Methods

### 2.1. Patient Selection Criteria

Children operated on for a posterior fossa pilocytic astrocytoma in the Pediatric Neurosurgery Department of the Catholic University Medical School, Rome, Italy were selected according to the following inclusion criteria:

(a) Age > 5 years (in order to have a complete set of neurocognitive evaluation data).
(b) No preventive treatment for hydrocephalus.
(c) The presence of a complete 10-year follow-up.

*Exclusion criteria*: (a) presence of severe neurological deficits, both at diagnosis and after surgery; (b) subarachnoid dissemination of the disease; (c) cerebellar mutism as an isolated clinical manifestation or as part of a posterior fossa syndrome.

All children with a PCF tumour that are admitted into our ward usually undergo a complete asset of neurocognitive evaluations. For this study, patients whose histological examinations revealed a pilocytic astrocytoma were selected. Among them, eight patients were excluded according to the aforementioned criteria. As stated before, neurocognitive evaluation is a key point in our usual PCF tumour management. Hence, no ethical committee approval was necessary for the present study.

### 2.2. Neuropsychological Assessment

In order not to bias the patients’ neurocognitive evaluation, for each round of tests, the examiners carried out a careful anamnesis to identify confounding factors. Neurocognitive functions were evaluated using the Wechsler Intelligence Scale, Third Edition (WISC III), which assesses the intellectual abilities of subjects with ages from 6 to 16 years and 11 months. This was performed in Italian and lasted 45 to 60 min, depending on each patient’s collaboration.

Global intellectual abilities were evaluated based on 3 scores: verbal IQ (VIQ), which is the sum of weighted scores of verbal subtests; performance IQ (PIQ), which is the sum of weighted performance subtest scores; and total IQ (TIQ), which is the sum of verbal and performance scores [7]. A mean TIQ value of 100 was considered normal, with an SD of 15. A difference of at least 12 points between the verbal IQ (VIQ) and performance IQ (PIQ) was considered statistically significant. 

Behaviour was tested using the Child Behavior Checklist (CBCL) [8]; this scale is a structured rating that asks caregivers (parents) to rate the child’s social and emotional problems at diagnosis or within the past 6 months. It is a set of 113 behavioural and emotional problems that parents rate using a 0 (not true), 1 (sometimes true) or 2 (very true) response set. The scale is suitable for students between 6 and 18. 

The CBCL results involve a three-way response: a total score, as well as two minor ones, namely, the internalising and externalising scores. The internalising score defines three narrow-band syndromes: anxious/depressed, withdrawn/depressed and somatic complaints. The externalising score defines the remaining 5 narrow-band syndromes, namely, social problems, thought problems, attention problems, rule-breaking behaviour and aggressive behaviour. The T score was used for the behavioural assessment evaluation (CBCL); a T score > 60 points according to the Achenbach criteria was considered abnormal [8].

“The bell test” was used in children aged 6 to 14 to evaluate attention processes with two types of scores: the speed score and the accuracy score [9]. The aforementioned test is a form of visual continuous performance task/cancellation task that is meant to evaluate selective attention and visuo-spatial abilities. The examiner has the children handle four different papers containing 35 bells each, as well as other animated and non-animated subjects. The patient has to select the bells in the fastest way possible after seeing the examiner performing the task on a sample sheet without knowledge of the time that is required to complete the task. The examiner will find out the speed at finding bells as well as the number of bells found after the test. The Leiter-R Battery was used to evaluate attention and executive function in children over 14 [9,10].

VMI (Visual–Motor Integration), a subtest to the Beery Test, was used to evaluate the child’s ability of visual and motor integration [11].

The Boston Naming Test was used to evaluate phonological, lexical, semantic, pragmatic and discursive skills [12].

*Time for testing*: All the aforesaid evaluations were performed (a) at diagnosis (T0), (b) 1 year after surgery (T1), (c) 5 years after surgery (T2) and (d) 10 years after the surgical treatment (T3).

### 2.3. Statistical Analysis

The authors used the Pearson and Spearman bivariate correlation to identify parameters correlating with the scores of neuropsychological tests. All the tests results were Z-normalised (mean value = 0, SD = 1). The individual Z-scores were then averaged to allow for a comparison between different tests and different groups of patients, where Z-score values < 1.66 were considered pathological.

## 3. Results

Thirty patients met the inclusion criteria (17 M/13 F; mean age at diagnosis: 6.80 years). No patient was on drugs for any kind of mental disorder before their admission. Nine children (9/30 = 31%) had a tumour involving the vermis and the right cerebellar hemisphere; in seven cases (7/30 = 23%), the tumour was localised at the level of the vermis and the left cerebellar hemisphere; in seven other children (7/30 = 23%), the tumour involved the IV ventricle and the right cerebellar hemisphere; and in the last seven cases (7/30 = 23%), the tumour extended from the IV ventricle to the left cerebellar hemisphere. Hydrocephalus was present at diagnosis in 14 cases (14/30 = 46%); 9 of them presented with moderate hydrocephalus, while 5 had severe hydrocephalus based on the MR Evans Index and the mammillo-pontine distance (Table 1). 

### 3.1. Preoperative Neurocognitive Evaluation (T0)

At diagnosis, thirteen children (13/30 = 43%) showed a borderline IQ (intelligence quotient (IQ): 80), with involvement of the coding skill and processing speed subtest in all cases. In seven of these thirteen cases (7/13 = 53%), the tumour was localised at the level of the fourth ventricle and right cerebellar hemisphere, while in three children, the tumour involved the fourth ventricle and the left cerebellar hemisphere (3/13 = 23%). Hydrocephalus was present in one child in each of these two sets of children (moderate in one and severe in the other). 

When neuropsychological functions were considered, twelve patients had difficulty in the area of language in terms of comprehension and lexical naming (12/30 = 40%), again with the tumour location at the level of the fourth ventricle and right cerebellar hemisphere (8/12 = 67%).

Visual-perceptive skills were involved in 16 cases (16/30 = 53%), with 7 of them having a tumour localisation at the level of the fourth ventricle and right cerebellar hemisphere (7/16 = 44%). Though hydrocephalus was present at diagnosis in 10 of these 16 children (moderate in 7, severe in 3 cases), there was no correlation with the evidence of a compromise in visual perceptive skills. 

Concerning behavioural aspects, eleven patients showed difficulties in the total score (11/30 = 36%), with five of them (45%) being affected by tumours involving the fourth ventricle and the right cerebellar hemisphere. 

When internalising scales were explored, seventeen children (17/30 = 56%) had anxiety and depression traits without a statistically significant correlation with tumour site and/or the presence or absence of active hydrocephalus, though an anxiety/depression profile was more frequently found in the group of children with fourth ventricle and right hemisphere locations (6/17 = 35%). 

Regarding the externalising scale, attention problems were documented in 10 children (10/30 = 33%), where 50 percent of them had a tumour located at the level of the fourth ventricle and right cerebellar hemisphere.

It is important to remember that the patients lacking in some areas at T0 were the same ones that maintained the deficits at later controls.

### 3.2. Postoperative Neurocognitive Evaluation after 1 Year (T1)

The first postoperative evaluation was carried out one year after surgery. Even though such a choice might be considered a limitation of the present study, we chose this time point in order to limit any possible interference from post-surgical manipulation. At T1, 12 of the 30 patients showed a borderline IQ (12/30 = 40%), with the persistence of manual and eye coordination difficulties; this result almost completely corresponded to the preoperative findings, both in absolute terms and in terms of the prevalent tumour location (the fourth ventricle and right cerebellar hemisphere were involved in more than 50% of the cases (7/12 = 58%). 

Significant variation was found in terms of the number of children presenting with an improvement in comprehension and lexical naming (8/30 = 26%, compared to 12/30 = 40% in the preoperative period); half of them, as before surgery, presented a tumour involving the fourth ventricle and the right cerebellar hemisphere.

No change was also evident in the number and selection of children with difficulties in visual-perceptive skills (15/30 = 50%, compared to 16/30 = 53% before surgery).

Concerning behavioural aspects, 33% (10/30) showed difficulties regarding the total score, a rate that almost completely corresponded to preoperative findings. 

When the internalising scale was explored, an increase was noted in the number of children with an anxiety and depression profile (20/30 = 66%, compared to 17/30 = 56%), which was a finding that did not correlate either with tumour location or with the presence of associated hydrocephalus.

When the externalising scale was explored, attention disorders identified before surgery persisted almost unchanged one year after the surgical treatment. 

### 3.3. Neurocognitive Evaluation after 5 Years (T2)

At the 5-year follow-up, no significant changes were noted in the percentage of children having a borderline IQ (10/30 = 33%), with forty percent of them being in the group of children with tumours localised at the level of the fourth ventricle and right cerebellar hemisphere. 

Similarly, vis-a-vis the preoperative and 1-year postoperative evaluation, lexical comprehension and naming were compromised in 10 cases (7/30 = 23%), and 15 cases (15/30 = 50%) still presented difficulties in visual perceptive skills. 

The results of the statistical analysis showed a single significant correlation in the assessment in comprehension and lexical naming for several patients.

We also observed a significant reduction in the number of children with anxiety/depression traits, with only nine children (9/30 = 30%) scoring below average in the behaviour profile. 

### 3.4. Neurocognitive Evaluation after 10 Years (T3)

Ten years after surgery, ten children (9/30 = 30%) still presented a borderline IQ, with 70% of them having been operated on for a tumour involving the fourth ventricle; there was no significant difference between those that had tumours extending to the right cerebella hemisphere and those with tumours extending to the left cerebellar hemisphere. 

Twenty percent (6/30) still presented comprehension and lexical naming difficulties, with around 60% of them harbouring tumours involving the IV ventricle and the right cerebellar hemisphere. 

We also observed a reduction in the number of children with difficulties in visual-perceptive skills (11/30 = 36%), with children that had fourth ventricle tumours representing the great majority (7/11 = 63%) of those with the persistence of such difficulties. 

**Figure 1:**
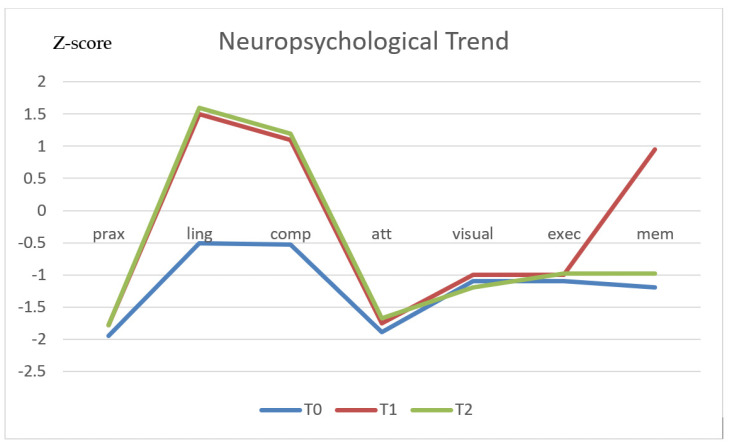
Neuropsychological functions.

A further significant improvement compared to the five-year follow-up evaluation was also noted in the scores of children with difficulties in language skills (Figure 1).

The results of behavioural testing remained stable, with no further sign of improvement noted in the group of children with anxiety/depression traits at 5 years. 

**Figure 2:**
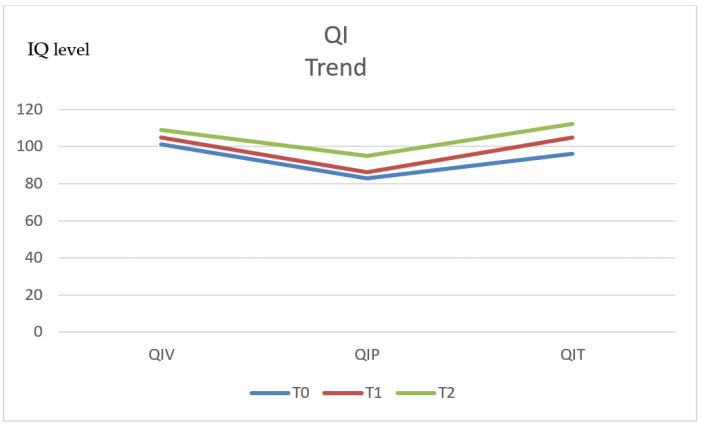
IQ trend.

In order to have further information about the influence of documented preoperative impairment of neurocognitive and behavioural functions, we also followed up children who had in-line neurocognitive acquisitions before surgery and scored normally when the behavioural tests were administered. These patients did not show any statistically significant worsening of their preoperative status after the tumour removal (Figure 2).

During the entire follow-up process, extra care was taken to ensure the absence of changes independent from the PCF tumour, with a consequent alteration in psycho-emotional conditions.

### 3.5. Statistical Analyses

Compared to the preoperative findings, we observed a significant improvement in the scores related to understanding skills at the evaluations performed after 1 year (p. 410*), which was confirmed after 5 years (p. 413*) and 10 years (p. 417*). A similar statistically significant improvement was documented regarding eloquence planning. The memory, visual perception and executive function test scores tended to progressively improve with successive follow-ups, but the related improvement did not reach statistical significance.

Praxic and visuo-perceptive skills also very moderately improved over time. 

Finally, taking into account the behavioural evolution over time, though the percentage of affected children was moderately reduced in terms of the total score after 1 (p. ,445*), 5 (p. ,528**) and 10 years (p. ,535**), the anxiety/depression trait scores did improve significantly at the evaluations performed after 1 (p. 415*), 5 (p. 440*) and 10 years (p. ,455* ), compared to the pre-operative condition, with coincidental improvements in the externalising scale scores after 1 (p. 416*), 5 (p. 517**) and 10 years (p. 525**) (Table 2).

Concerning the tumour locations, there was a statistically significant correlation between the presence of neurocognitive and behavioural deficits and the location of the tumour at the level of the fourth ventricle extending to the right cerebellar hemisphere. This correlation was confirmed during the follow-ups at 1, 5 and 10 years. In contrast, no correlation was found between hydrocephalus at admission and neurocognitive impairment, both at admission and during the follow-ups. 

**Figure 3:**
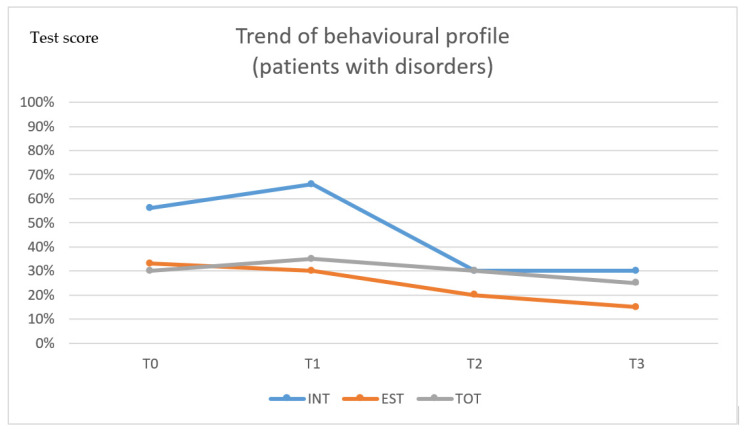
Trend of behaviour.

When selective skills were separately evaluated, the most significantly improved functions were expressive and receptive language skills, whereas only mild improvements were observed in visual perception and memory, praxis and attention. Regarding behavioural aspects, a significant improvement in the scores was observed in all three spheres of behaviour investigated by the instrument, although it appeared to be more evident in males than in females (Figure 3).

## 4. Discussion

Forty-three percent of the children selected for the present study showed a borderline IQ before surgery. Such a finding is concordant with the percentage reported in the literature about children that are affected by posterior fossa pilocytic astrocytomas [12]. As previously described, praxis and visual perception were the selective functions that were more frequently affected, with VMI scores being the overall lowest ones compared with the other selective test scores [6].

Language performance test scores were below average in 40% of the cases and tended to improve in terms of expressive and receptive skills, even at the 1-year follow-up. Regarding further follow-ups, the aforementioned improvement became significant at the 5-year follow-up (*p* < 0.5) and 10-year follow-up (*p* < 0.5). The benign nature of the tumour, mean presentation age and early rehabilitation protocol were considered key factors in positively influencing the children’s recovery in terms of language skills [13,14,15].

The benign nature of the tumour was deemed important, as it allowed children to concentrate on rehabilitation protocol without intervals due to adjuvant treatment. Further, by avoiding adjuvant therapies, related adverse events were also averted. Regarding our samples, the low mean age was considered beneficial, thanks to the higher recovery abilities. Nonetheless, the early rehabilitation protocols were undoubtedly the major factor responsible for improvement. The above-mentioned rehabilitation protocol was tailored for each patient according to the personal lack of skills and consisted mainly of psychomotricity and/or logopedy. Rehabilitation usually started one month after surgery, including procedures that were aimed at improving sentence structure, word structure, concepts and directions, formulated sentences, word classes, recalling sentences, sentence assembly, semantic relationships, word associations, listening to paragraphs, rapid, automated naming and working memory [16,17,18,19].

Praxic and visuo-perceptive skills tended to be more resistant to this rehabilitation plan, as documented by the slight improvement observed in 5 of the 16 children with defective results before surgery [20,21].

These results were linked to the higher complexity of the circuits involved in these functions, and the prominent role of the brainstem tracts and dominant (right) cerebellar side in the affected children. Alterations of the dominant side’s sensorimotor processing may have indeed led patients in the present series to rely only on visuo-spatial information to process bodily images, rather than relying on a combination of visuo-spatial information with sensorimotor processing. Our results also suggest that the development of mental transformation and motor execution abilities reflected the refinement of a higher-order internal representation of actions [22].

Based on the previously described evidence, we suggest submitting affected children to an early integrated rehabilitation program that includes visual-spatial stimulation. Including praxic and visuo-perceptive skill rehabilitation tasks ab initio is important to exploit the greater benefits of these skills that can be gained in the first five years after surgery. Further, since these skills are harder to rehabilitate, the earlier the exercise is commenced, the better [21,23].

When behavioural aspects were considered, the lack of interest and outsourcing tended to significantly improve during follow-up, whereas anxiety and depression traits tended to improve only moderately.

Commonly persisting behaviours that were observed by us included distractibility, hyperactivity, impulsiveness, disinhibition, anxiety, illogical thought and lack of empathy, as well as aggression and irritability [13,24,25]. Further, other traits found were: ruminative and obsessive behaviours, dysphoria and depression, tactile defensiveness, sensory overload, apathy and the inability to appreciate social boundaries. Targeted behavioural therapy should be combined with physical and speech rehabilitation programs, as improvement in motor and speech strongly influence the rate of behavioural improvement. 

As previously mentioned, it is worth noting that, in our cohort, all neurocognitive alterations highlighted during the follow-ups were found in children who presented the same disturbances even at diagnosis, while children with preoperative scores in line with their chronological age did not show a worsening of their cognitive status after the tumour removal. Two considerations emerge from this result: (1) Preoperative neurocognitive evaluation, despite an apparently normal cognitive status of the child, was of paramount importance since it was predictive of the postoperative course. Such a consideration is very important in order to give correct information to the parents about what can be expected after surgery, as well as informing them of the related need for a rehabilitation program. (2) Surgical treatment did not represent a risk factor for child neurocognitive development, whereas tumour location had a relevant role in this context. 

Unlike what has been previously reported, the presence of hydrocephalus did not seem to influence the results in our group of children. This was probably related to the fact that, though considered active on preoperative MR imaging, ventricular dilation was moderate in terms of its severity in the majority of the cases (9/14 cases = 64%).

Finally, it is important to underline how, in our study, only patients without the appearance of serious neurological deficits pre- or post-operatively were studied. This fact might be considered a limitation of our work and depended on the intent in predicting the neuropsychological outcome. In fact, such disturbance factors would have made it practically impossible to suggest the future outcome from the incredibly high number of variables involved. Further studies are warranted in order to characterise such conditions. 

## 5. Conclusions

Recognising and measuring both the short- and long-term effects of cerebellar tumours in children and their treatment is the first step towards improving their clinical course and their quality of life. Early recognition of the disorders is essential for educating families about what to expect regarding the clinical course, cognitive and physical recovery and timing for possible improvement [26]. Early interventions should be provided to all patients facing this condition, with specific attention to visual-spatial stimulation, speech and occupational therapies (in that order); it is important to act on praxic and visuo-perceptive skill, as well as on emotion and behaviour, tracts of the neurocognitive profile in the beginning since they more commonly tend to persist in the long term. 

## Figures and Tables

**Table 1:** Patients’ characteristics.

Mean Age at Diagnosis	6.80 Years
Sex	17 M/13 F
Hydrocephalus	14/30 patients
Lateralisation	14 left, 16 right
Localisation	16 vermis, 14 IV ventricle

**Table 2:** Mean values and standard deviations.

	T0		T1		T2	
	Mean	SD	Mean	SD	Mean	SD
Prax	−1.95	0.91	−1.78	0.75	−1.78	0.75
Ling	−0.51	1.6	1.5	1.02	1.6	0.99
Comp	−0.53	1.59	1.1	1.54	1.2	1.52
Att	−1.89	0.99	−1.75	0.79	−1.68	0.65
Visual	−1.1	1.25	−1	1.34	−1.2	1.29
Exec	−1.1	1.45	−1	1.45	−0.98	1.68
Mem	−1.2	0.95	−1.02	0.95	−0.98	1.15
VIQ	101	19	105	18	109	15
PIQ	83	23	86	19	95	17
TIQ	96	17	105	14	112	11
INT	70	7	65	5	62	3
EST	65	6	58	4	55	4
TOT	68	7	56	5	54	3

## Data Availability

Not applicable.
